# Postoperative myopic shift and visual acuity rehabilitation in patients with bilateral congenital cataracts

**DOI:** 10.3389/fmed.2024.1406287

**Published:** 2024-05-02

**Authors:** Duoru Lin, Qiaolin Zhu, Shuyi Zhang, Fengqi Zhou, Lanqin Zhao, Qiwei Wang, Wan Chen, Hui Chen, Xiaoshan Lin, Huanling Feng, Qiuping Zhong, Jingjing Chen, Zhuoling Lin, Xiaoyan Li, Wei Xiao, Yue Zhou, Jinghui Wang, Jing Li, Weirong Chen

**Affiliations:** ^1^State Key Laboratory of Ophthalmology, Zhongshan Ophthalmic Center, Sun Yat-sen University, Guangdong Provincial Key Laboratory of Ophthalmology and Visual Science, Guangdong Provincial Clinical Research Center for Ocular Diseases, Guangzhou, China; ^2^Mayo Clinic College of Medicine and Science, Rochester, MN, United States; ^3^Department of Ophthalmology, Mayo Clinic Health System, Eau Claire, WI, United States

**Keywords:** bilateral congenital cataract, myopic shift, spherical equivalent, visual acuity rehabilitation, cataract surgery

## Abstract

**Background:**

This study aimed to explore the postoperative myopic shift and its relationship to visual acuity rehabilitation in patients with bilateral congenital cataracts (CCs).

**Methods:**

Bilateral CC patients who underwent cataract extraction and primary intraocular lens implantations before 6 years old were included and divided into five groups according to surgical ages (<2, 2–3, 3–4, 4–5, and 5–6 years). The postoperative myopic shift rates, spherical equivalents (SEs), and the best corrected visual acuity (BCVA) were measured and analyzed.

**Results:**

A total of 1,137 refractive measurements from 234 patients were included, with a mean follow-up period of 34 months. The postoperative mean SEs at each follow-up in the five groups were linearly fitted with a mean *R*^2^ = 0.93 ± 0.03, which showed a downtrend of SE with age (linear regression). Among patients with a follow-up of 4 years, the mean postoperative myopic shift rate was 0.84, 0.81, 0.68, 0.24, and 0.28 diopters per year (D/y) in the five age groups (from young to old), respectively. The BCVA of those with a surgical age of <2 years at the 4-year visit was 0.26 (LogMAR), and the mean postoperative myopic shift rate was 0.84 D/y. For patients with a surgical age of 2–6 years, a poorer BCVA at the 4-year visit was found in those with higher postoperative myopic shift rates (*r* = 0.974, *p* = 0.026, Pearson’s correlation test).

**Conclusion:**

Performing cataract surgery for patients before 2 years old and decreasing the postoperative myopic shift rates for those with a surgical age of 2–6 years may benefit visual acuity rehabilitation.

## 1 Introduction

Congenital cataract (CC) is an ocular abnormality of lens opacity that exists at birth or gradually forms early after birth and has become the leading cause of childhood blindness (1). Timely surgery in the critical period of visual development to relieve the form deprivation caused by the opaque lens can reduce irreversible visual impairments (2). With the advancement of surgical techniques and skills and anesthesia techniques in recent years, it is not difficult for pediatric ophthalmologists to perform cataract extraction and intraocular lens (IOL) implantation in patients of any age, even very young infants. However, the postoperative myopic shift remains a challenge for visual reconstruction and could increase the risk of high myopia complications (3). Understanding the distribution of postoperative refractive change in patients of different surgical ages is clinically significant for reducing myopic shift. Researchers have investigated the myopic shift of patients with unilateral CC who need to undergo surgery as early as possible after detection to reduce the risk of visual development inhibition (amblyopia) caused by form deprivation in the affected eyes (4–6). However, due to the less obvious binocular visual competition and inhibition, the findings from unilateral CC patients could not apply to bilateral CC patients. The visual acuity and postoperative myopic shift of bilateral CC patients have been previously reported (7, 8), but the interrelation between them remained unclear, which might be due to the insufficient follow-up period or small cohort. In this study, we aimed to explore the postoperative myopic shift after cataract extraction and primary IOL implantation and its relation to visual acuity rehabilitation in patients with bilateral CCs. The findings may provide a reference for the postoperative myopic shift management and visual rehabilitation of Chinese children with bilateral CCs, a special population that is more likely to develop myopia (9).

## 2 Materials and methods

### 2.1 Subjects and methods

Patients with CC were retrospectively enrolled in the Childhood Cataract Program of the Chinese Ministry of Health (CCPMOH), Zhongshan Ophthalmic Center (ZOC), Guangzhou, China, a longitudinal, observational study that was designed to reduce childhood blindness and visual impairment caused by CC. This study was approved by the Ethics Committee of ZOC at Sun Yat-sen University (No. 2020KYPJ149) and was conducted in accordance with the Declaration of Helsinki. Informed written consent was obtained from at least one parent of each patient. The inclusion criteria were set as follows: (1) diagnosed with bilateral CCs (lens opacities in both eyes present at birth or within 1 year after birth) according to the morphology of lens opacities and patient history; (2) with severe visual impairment that required surgical treatment; (3) underwent cataract extraction and primary IOL implantation at the ZOC between December 2010 and June 2018; and (4) with a surgical age under 6 years old. Patients with a follow-up period of fewer than 6 months were excluded. Patients with other ocular abnormalities, such as microphthalmos, microcornea, aniridia, and persistent fetal vasculature, were also excluded. This study followed the Strengthening the Reporting of Observational Studies in Epidemiology (STROBE) reporting guidelines (10).

### 2.2 Surgical procedures

All surgeries were performed by two experienced pediatric ophthalmologists (YZL and WRC). Anterior capsulotomy was performed in a continuous curve. The nucleus and cortex were removed using an irrigation/aspirating handpiece. Posterior capsulotomy and limited anterior vitrectomies were performed in children aged 6 years or younger (11). The axial length was measured preoperatively by contact A-scan ultrasound (B-SCAN-Vplus/BIOVISION, Quantel Medical, France), and the SRK-T formula was used to calculate the IOL power. The refractive targets were set with reference to the practice styles and preferences of the American Society of Cataract and Refractive Surgery (ASCRS) and the American Association for Pediatric Ophthalmology and Strabismus (AAPOS) members, ranging from +6D to +1D according to the patient’s age (12, 13). The AcrySof SA60AT, SN60AT, and MA60AC IOLs (Alcon Laboratories, Fort Worth, TX, USA) were implanted in the capsular bag.

### 2.3 Follow-up and measurements

All patients have required follow-ups at 1 month, 3 months, 6 months after surgery, and every half year thereafter. The postoperative spherical equivalent (SE) and best corrected visual acuity (BCVA) were recorded at each follow-up. The last follow-up was set as the fourth year (±3 months) visit after surgery. The myopic shift rate was defined as the change of SE between the first and fourth year visits divided by the time interval. Subjective refraction was performed by a certified optometrist. Young patients who were unable to cooperate underwent cycloplegic retinoscopy after sedation with 10% chloral hydrate (0.8 mL/kg, oral or rectal administration) (14). The pupils were dilated with 0.5% compound tropicamide eye drops (Zhuo Bi’an, Xingqi Eye Medicine Company Limited, China) before slit-lamp and refractive examination (usage: 3 times, one drop every 5 min). Refractions were recorded as SE, calculated by spherical power and cylindrical power for each eye (algebraic sum in diopters (D), sphere +1/2 cylinder). Spectacles were prescribed, and the patients were required to wear glasses at all times. Guidance for wearing the glasses was provided at every follow-up. The posterior capsular opacity (PCO) was evaluated at every follow-up, and the patients with moderate to severe VAO underwent timely YAG laser capsulectomy to reduce the influence on BCVA evaluation.

### 2.4 Statistical analysis

All data were entered into Microsoft Excel (Microsoft Corp., Redmond, Washington, USA) spreadsheets, sorted, and analyzed by three researchers (SYZ, DRL, and QLZ). The data were further imported into the Statistical Package for the Social Sciences (SPSS ver. 19.0, Chicago, IL, USA) for statistical analysis. All included patients were divided into five groups according to their surgical ages: <2 years, 2–3 years (≥2 and < 3 years), 3–4 years (≥3 and < 4 years), 4–5 years (≥4 and < 5 years), and 5–6 years (≥5 and < 6 years). Linear regression was used to show the variational trends of the postoperative myopic shift among the five groups. Among patients with a follow-up of 4 years, the myopic shift rate, SE, and BCVA of patients in the five groups at the 4-year visit were compared using generalized estimating equations (GEEs) with robust standard errors to adjust for the correlation between the two eyes, sex, and baseline SE, and Bonferroni was adopted for the pairwise multiple comparisons. The relationship between the postoperative myopic shift rates and BCVA (using the mean value of both eyes) at the 4-year visit was evaluated using Pearson’s correlation test. Patients who did not complete the 4-year follow-up were defined as lost to follow-up. The surgical age was compared using Student’s t-test, while the baseline SE and baseline BCVA were compared using GEE between patients who lost follow-up and those who did not. All statistical tests were two-tailed, and the level of significance was set at 0.05.

## 3 Results

**Figure 1:**
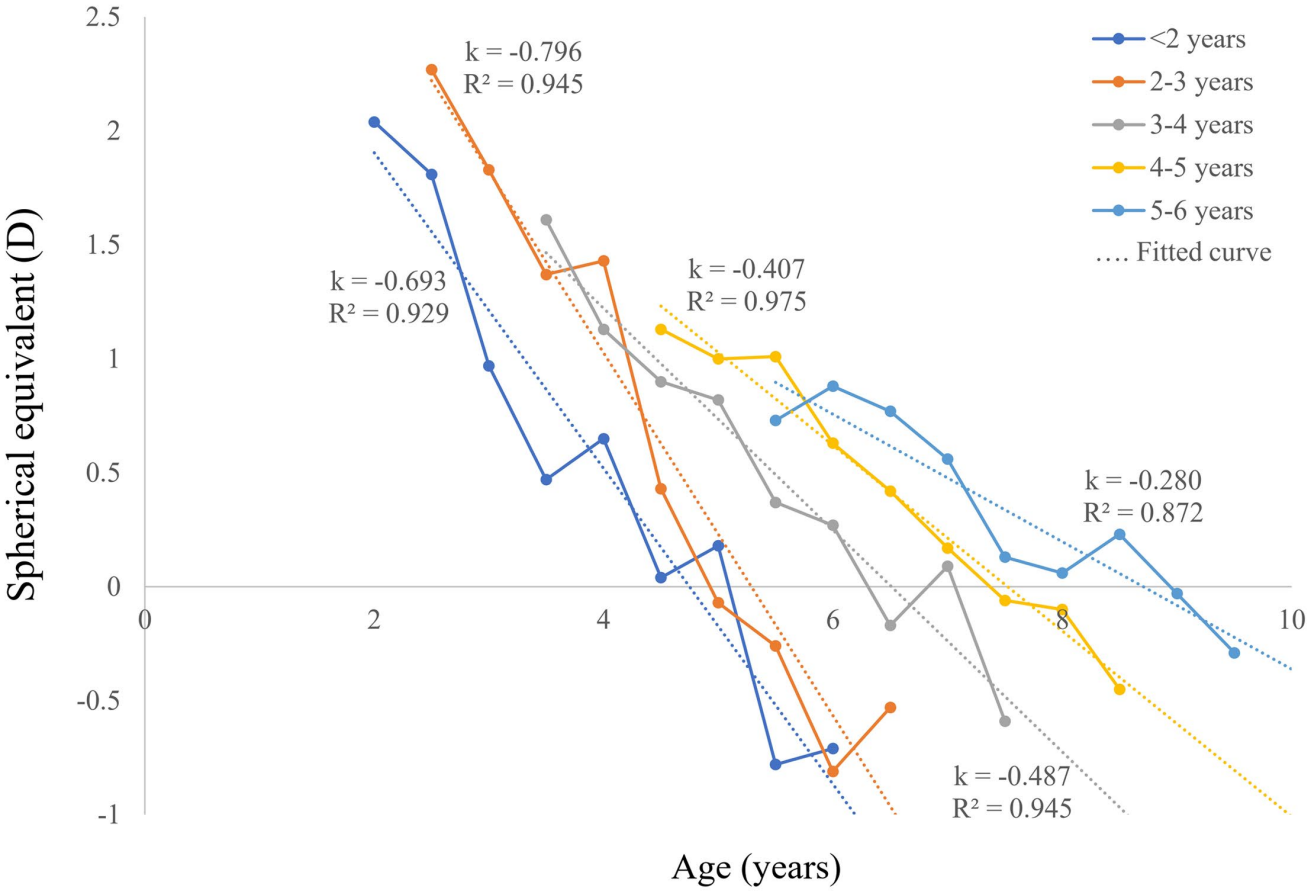
Postoperative SE and linear fitted lines of bilateral CC patients with different surgical ages. CC, congenital cataract; SE, spherical equivalent; D, diopters.

A total of 1,137 refractive measurements from 234 patients with bilateral CCs were included. The patient characteristics of the five groups are shown in Table 1. The mean follow-up period of all patients was 34 months (range, 7–52 months, including those lost to follow-up). The postoperative SEs at each follow-up visit of patients in the five groups are presented in Table 2. To show the variation trends of postoperative myopic shift, the mean SEs of each follow-up were linearly fitted in Figure 1 (including those lost to follow-up), with a mean *R*^2^ value of 0.931 ± 0.03. All fitted lines showed a downtrend in refractive error with age. According to the fitted lines, the mean ages of emmetropia (SE equals zero) were 4.95, 5.09, 6.20, 7.44, and 8.08 years in the five groups with different surgical ages (from young to old).

**Table 1:** Patient characteristics in the five groups with different surgical ages.

Surgical age	< 2 years	2–3 years	3–4 years	4–5 years	5–6 years
Patients	18	48	74	56	38
Refractions	101	266	399	288	211
Sex (male: female)	14:4	27:21	47:27	34:22	21:17
Age at surgery (months)					
Mean (SD)	19.56 (3.09)	29.52 (3.87)	41.00 (3.46)	53.61 (3.26)	65.97 (3.43)
Range	13, 23	24,35	36,47	48,59	60,71
IOL power (D)					
Mean (SD)	22. 87 (3.06)	22.43 (4.50)	22.94 (4.26)	22.44 (5.00)	22.21 (4.65)
Range	16.0, 28.5	11.5, 32.0	13.5, 32.0	8.0, 34.0	9.5, 32.0
Follow-up (months)^#^					
Mean (SD)	39.5 (15.3)	38.9 (11.0)	38.4 (10.6)	35.1 (12.3)	36.2 (13.8)
Range	8.1, 58.7	9.5, 55.6	12.0, 54.3	8.5, 51.7	7.1, 53.9
Age at the 4-year follow-up (months)^#^					
Mean (SD)	60.0 (12.6)	68.9 (11.3)	79.9 (9.6)	91.7 (12.8)	102.6 (13.3)
Range	29.8, 72.5	34.7, 80.9	54.4, 92.6	58.8, 104.7	75.7,116.9

^#^Including those lost to follow-up; SD, standard deviation; IOL, intraocular lens; D, diopter.

**Table 2:** Postoperative SE of patients in the five groups with different surgical ages.

Surgical age: < 2 years	Age at follow-up, years	2	2.5	3	3.5	4	4.5	5	5.5	6
	SE, D Mean (SD)	2.04 (1.35)	1.81 (1.30)	0.97 (0.96)	0.47 (1.14)	0.65 (1.48)	0.04 (1.55)	0.18 (1.40)	−0.78 (0.92)	−0.71 (1.20)
	Range, D	−0.56, 4.63	0.06, 4.06	−0.38, 2.50	−0.94, 2.25	−1.43, 2.63	−1.94, 2.31	−1.75, 2.06	−2.31, 0.69	−2.19, 1.06
	95% CI, D	1.31, 2.76	1.06, 2.56	0.23, 1.71	−0.25, 1.20	−0.29, 1.59	−1.05, 1.13	−1.29, 1.65	−1.73, 0.17	−1.97, 0.55
	Patients	16	14	9	12	12	11	6	8	6
Surgical age: 2–3 years	Age at follow-up, years	2.5	3	3.5	4	4.5	5	5.5	6	6.5
	SE, D Mean (SD)	2.27 (1.78)	1.83 (1.91)	1.37 (1.83)	1.43 (1.74)	0.43 (1.45)	−0.07 (1.65)	−0.26 (1.41)	−0.81 (1.42)	−0.53 (1.46)
	Range, D	−0.25, 5.75	−0.63, 7.06	−1.00, 5.38	−0.88, 4.69	−1.25, 4.06	−2.38, 4.06	−2.88, 2.44	−3.44, 1.94	−4.19, 1.81
	95% CI, D	1.50, 3.04	1.16, 2.50	0.69, 2.05	0.76, 2.10	−0.12, 0.98	−0.75, 0.61	−0.92, 0.40	−1.42, −0.20	−1.21, 0.16
	Patients	23	34	30	28	29	25	20	23	20
Surgical age:3–4y	Age at follow-up, years	3.5	4	4.5	5	5.5	6	6.5	7	7.5
	SE, D Mean (SD)	1.61 (1.96)	1.13 (1.69)	0.90 (2.19)	0.82 (2.03)	0.37 (1.74)	0.27 (2.02)	−0.17 (2.19)	0.09 (2.34)	−0.59 (2.50)
	Range, D	−2.50, 7.50	−2.81, 7.38	−5.63, 7.81	−5.19, 7.75	−4.50, 4.25	−4.19, 6.56	−4.69, 6.31	−4.75, 6.13	−6.94, 2.75
	95% CI, D	1.00, 2.23	0.65, 1.61	0.30, 1.50	0.22, 1.43	−0.19, 0.94	−0.33, 0.87	−0.95, 0.61	−0.80, 0.98	−1.73, 0.55
	Patients	41	50	53	46	39	46	33	29	21
Surgical age: 4–5 years	Age at follow-up, years	4.5	5	5.5	6	6.5	7	7.5	8	8.5
	SE, D Mean (SD)	1.13 (1.82)	1.00 (1.14)	1.01 (1.59)	0.63 (1.27)	0.42 (1.70)	0.17 (1.09)	−0.06 (1.24)	−0.10 (1.23)	−0.45 (2.16)
	Range, D	−1.69, 7.31	−1.06, 4.00	−1.31, 7.00	−1.38, 3.69	−2.19, 7.56	−2.75, 1.56	−2.50, 2.31	−2.88, 2.13	−5.19, 6.13
	95% CI, D	0.49, 1.76	0.64, 1.36	0.44, 1.58	0.18, 1.09	−0.23, 1.06	−0.28, 0.61	−0.57, 0.45	−0.64, 0.43	−1.40, 0.51
	Patients	34	41	32	32	29	26	25	23	22
Surgical age: 5–6 years	Age at follow-up, years	5.5	6	6.5	7	7.5	8	8.5	9	9.5
	SE, D Mean (SD)	0.73 (1.44)	0.88 (1.53)	0.77 (1.40)	0.56 (1.47)	0.13 (1.80)	0.06 (1.86)	0.23 (1.57)	−0.03 (2.02)	−0.29 (1.99)
	Range, D	−1.63, 3.69	−1.56, 4.19	−1.75, 3.19	−2.75, 3.69	−3.69, 3.38	−4.19, 3.75	−1.75, 3.50	−4.38, 3.50	−4.06, 3.13
	95% CI, D	0.07, 1.38	0.33, 1.43	0.22, 1.31	−0.06, 1.18	−0.71, 0.97	−0.93, 1.05	−0.65, 1.10	−0.88, 0.82	−1.43, 0.85
	Patients	21	32	28	24	20	16	15	19	14

SE, spherical equivalent; D, diopters; SD, standard deviation; CI, confidence interval.

**Figure 2:**
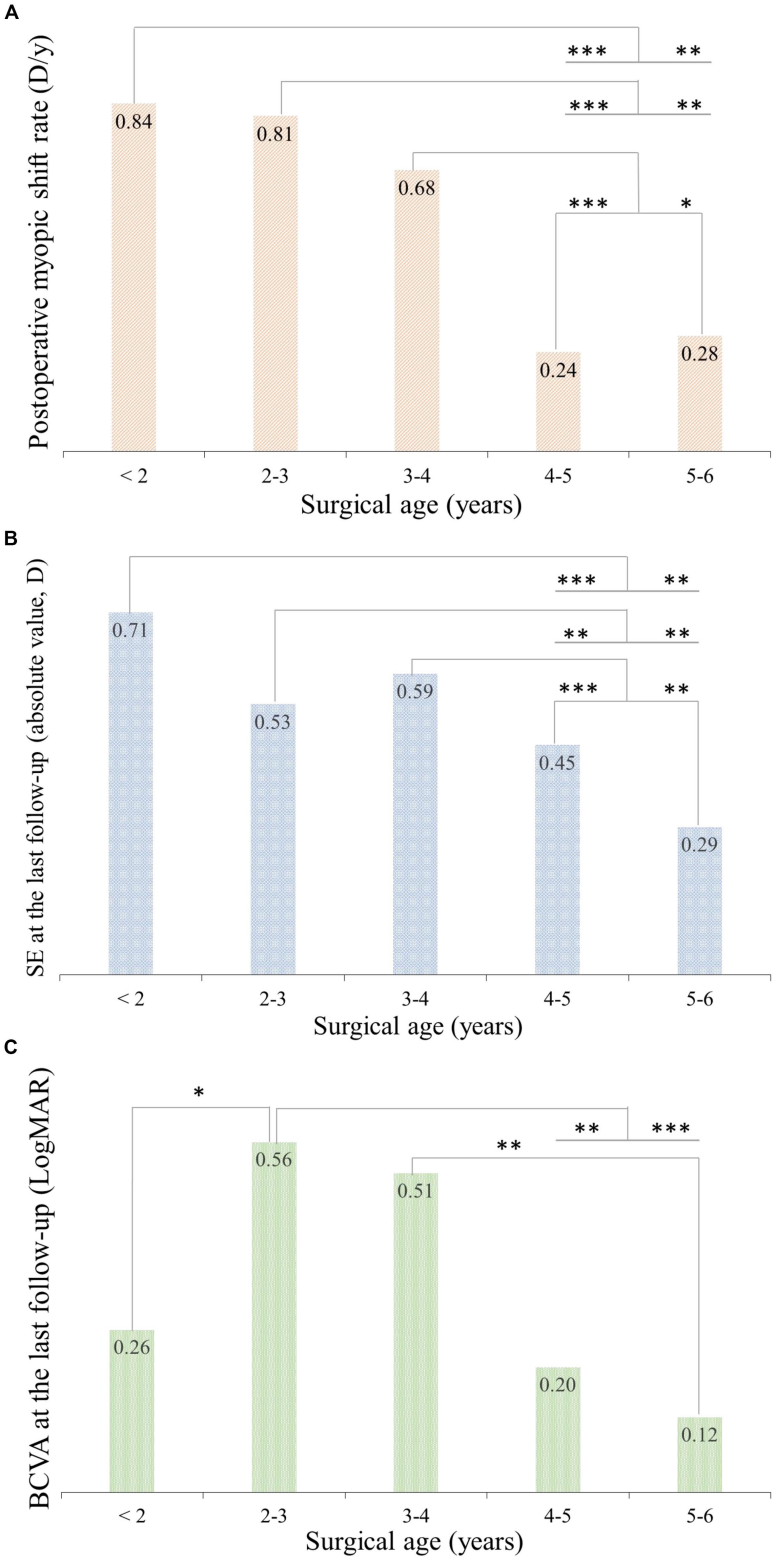
Comparisons of mean postoperative myopic shift rate, SE, and BCVA at the 4-year visit among patients with different surgical ages. **(A)** Significant differences in mean postoperative myopic shift rate in patients with different surgical ages were revealed (*p* < 0.001, adjusting the correlation between two eyes, sex, and baseline SE using GEE). Significant differences in mean SE **(B)** and BCVA **(C)** were also found among the five groups (*p* < 0.001, adjusting the correlation between two eyes, sex, and baseline SE using GEE). SE, spherical equivalent; BCVA, best corrected visual acuity; D, diopters; D/y, diopter per year; *p < 0.01, **p < 0.005, ***p ≤ 0.001.

As shown in Figure 2A, the postoperative myopic shift rate of each group was 0.84 (standard error: 0.22), 0.81 (0.53), 0.68 (0.55), 0.24 (0.34), and 0.28 (0.38) diopters per year (D/y) in patients with surgical ages of <2, 2–3, 3–4, 4–5, and 5–6 years, respectively. Significant differences in the postoperative myopic shift rates among patients with different surgical ages were revealed (*p* < 0.001, adjusting for the correlation between two eyes, sex, and baseline SE using GEE). Specifically, the postoperative myopic shift rates of patients with surgical ages of <2 years were larger than those older than 4 years (p < 0.001 in patients with surgical ages of 4–5 years, *p* = 0.005 in patients with surgical ages of 5–6 years). Similarly, the postoperative myopic shift rates of patients with surgical ages of 2–3 years and patients with surgical ages of 3–4 years were larger than those older than 4 years, respectively (*p* = 0.001, *p* = 0.007, *p* = 0.001, and *p* = 0.012).

Significant differences in the mean SE were found at the 4-year visit among the five groups (*p* < 0.001, adjusting for the correlation between two eyes, sex, and baseline SE using GEE, Figure 2B). Specifically, patients with surgical ages of <2 years, 2–3 years, and 3–4 years were more myopic than those older than 4 years, respectively (*p* = 0.001 and *p* = 0.007, *p* = 0.002 and *p* = 0.007, *p* = 0.001 and *p* = 0.007).

The mean BCVA at the 4-year visit in each group is presented in Figure 2C, with significant differences among patients with varying surgical ages (*p* = 0.005, adjusting the correlation between two eyes, sex, and baseline SE using GEE). Specifically, patients with a surgical age of 2–3 years had worse BCVA than those with a surgical age of <2 years (*p* = 0.013), 4–5 years (*p* = 0.008), and 5–6 years (*p* = 0.001). Patients with a surgical age of 3–4 years had worse BCVA than those with a surgical age of 5–6 years (*p* = 0.007).

The mean postoperative myopic shift rate, SE, and BCVA at the 4-year visit among patients with different surgical ages were comprehensively analyzed. Even though the mean postoperative myopic shift rates of patients with a surgical age of <2 years reached up to 0.84 D/y, their mean BCVA at the 4-year visit was 0.26 in LogMAR. For those with a surgical age of 2–6 years, the higher the postoperative myopic shift rate, the poorer the BCVA at the 4-year visit (*r* = −0.974, *p* = 0.026, Pearson’s correlation test). Furthermore, for those with a surgical age of 2–6 years, the older the surgical age, the better BCVA at the 4-year visit (*r* = −0.957, *p* = 0.043, Pearson’s correlation test).

To reduce the potential bias caused by the patients’ loss to follow-up in the later periods, we compared the patient characteristics, postoperative SE, and BCVA at the 6-month follow-up between patients who lost to follow-up and those who did not. No significant difference was found (Supplementary Table).

## 4 Discussion

In this study, we provided a description of the postoperative myopic shift by age group, visual acuity rehabilitation, and their relationships in a total of 234 bilateral CC patients who underwent cataract extraction and primary IOL implantations at less than 6 years old. A higher rate of postoperative myopic shift was found in patients with younger surgical ages. For patients with surgical ages of 2 to 6 years, a poorer BCVA was found in those with higher postoperative myopic shift rates. However, the BCVA of patients with a surgical age of <2 years at the 4-year visit was acceptable, even though their postoperative myopic shift rates were high.

We found that a higher rate of the postoperative myopic shift was found in patients with younger surgical ages, which is similar to the findings of other studies (7, 15–17). For example, Astle et al. (17) included both unilateral and bilateral CC patients and divided them into four groups according to surgical age (<2, 2–4, 4–7, and 7–18 years). They found that the postoperative myopic shift rate was high in patients under 4 years old (−1.85, −1.10, −0.64, and − 0.30 D/y, respectively). The eyeball grows fastest during young age (2), and we suspected that performing cataract extraction and primary IOL implantation with planned under-correction of hyperopia in children at a younger age are more likely to lead to non-physiologically hyperopic defocus and abnormal growth of the visual axis, presenting a larger myopic shift (18).

Postoperative BCVA is another important factor for postoperative visual rehabilitation. The results showed that for patients with surgical ages of 2 to 6 years, the higher the postoperative myopic shift rates, the poorer the BCVA at the 4-year visit. A larger defocus caused by the myopia shift could contribute to severe amblyopia, resulting in poorer postoperative BCVA (19). Therefore, for CC patients who are detected later in life and undergo surgery between 2 and 6 years, reducing the postoperative myopic shift may be beneficial for visual acuity rehabilitation. Additionally, it was found that older surgical age was associated with better BCVA at the 4-year visit in these patients, which is inconsistent with some previous studies (20, 21). Some of these patients might have mild cataracts in the initial stage, and mild form deprivation may not have seriously affected the visual development at the critical stage. The lens opacities gradually worsened and affected vision after that, and therefore, these patients were detected and operated on at an older age. In general, the postoperative visual acuity of these patients may improve, although at older surgical ages (22). Future studies are warranted to verify these findings and the possible interpretations.

For patients with surgical ages <2 years, our data showed that even though the postoperative myopic shift rates were high, their BCVAs at the 4-year visit were relatively acceptable. Visual input in the early stage of life is necessary for visual development, and early visual deprivation can cause later deficits in the visual system, especially for those younger than 2 years (23). Although the myopic shift rate is high, early removal of the clouded lens with refractive correction is still beneficial for the recovery of visual function. Therefore, early detection of vision-threatening CCs and timely surgery before the end of the critical stage of visual development may be one of the key factors for the visual rehabilitation of patients with bilateral CCs.

There are some limitations in this study. First, the findings are only applicable to patients with bilateral CCs who underwent cataract removal and primary IOL implantation before 6 years of age, and its postoperative follow-up period was only up to 4 years. An extension to patients with different surgical ages, follow-up periods, and surgical approaches should be interpreted with caution. In addition, although we presented the variation trend of the postoperative myopic shift in a four-year follow-up period, the numbers of young patients and those in the later follow-up periods were relatively small (maybe due to the COVID-19 prevention policy and other reasons). However, no significant difference in patient characteristics, SE, or BCVA was found between patients with follow-up and those without in the 6-month follow-up. Furthermore, even though family history, morphological features, and even full exon sequencing results were used to confirm the CC diagnosis, a small portion of developmental cataracts may be inevitably included. Finally, this study mainly focused on postoperative refractive changes, visual acuity, and their relationships. The potential factors affecting postoperative refractive changes and visual acuity rehabilitation will be included and analyzed in our next study.

In conclusion, this study with a large sample size and long follow-up period (up to 4 years) explored the myopic shift and its relationship with visual acuity rehabilitation in patients with bilateral CCs. Among patients with a surgical age under 6 years, a higher rate of postoperative myopic shift was found in patients with younger surgical ages. Performing cataract surgery for patients younger than 2 years and decreasing the postoperative myopic shift rates for those with a surgical age of 2–6 years may be beneficial to visual acuity rehabilitation. These findings may provide a reference for the postoperative myopic shift management and visual rehabilitation of children with bilateral CCs.

## Data availability statement

The original contributions presented in the study are included in the article/Supplementary material, further inquiries can be directed to the corresponding authors.

## Ethics statement

The studies involving humans were approved by the Ethics Committee of ZOC at Sun Yat-sen University. The studies were conducted in accordance with the local legislation and institutional requirements. The participants provided their written informed consent to participate in this study.

## Author contributions

DL: Conceptualization, Funding acquisition, Methodology, Project administration, Supervision, Writing – original draft, Writing – review & editing. QZhu: Data curation, Project administration, Writing – original draft. SZ: Writing – original draft, Writing – review & editing, Formal analysis, Software, Validation. FZ: Writing – review & editing. LZ: Writing – review & editing, Formal analysis, Software. QW: Writing – review & editing. WaC: Writing – review & editing. HC: Writing – review & editing. XLin: Validation, Writing – review & editing. HF: Writing – review & editing. QZho: Writing – review & editing. JC: Writing – review & editing. ZL: Writing – review & editing. XLi: Writing – review & editing. WX: Writing – review & editing. YZ: Writing – review & editing. JW: Writing – review & editing. JL: Writing – review & editing. WeC: Project administration, Resources, Supervision, Writing – review & editing.
