# Supplementary material for: Postoperative myopic shift and visual acuity rehabilitation in patients with bilateral congenital cataracts
Source: Front Med (Lausanne). 2024 May 2;11:1406287. doi: 10.3389/fmed.2024.1406287 (PMC11096542; doi:10.3389/fmed.2024.1406287)
Supplement: Supplementary file 1 [file Table_1.DOCX]

**Supplementary Table. Comparisons of characteristics, SE and BCVA at the first visit between patients with follow-up and those lost to follow-up.**

| **Surgical age:**  **< 2 y** |  | With follow-up | Lost to follow-up# | P value |
| --- | --- | --- | --- | --- |
|  | Patients | 12 | 6 | NA |
|  | Sex (M: F) | 8:4 | 6:0 | 0.162 |
|  | Age at surgery, mo  Mean(SD) | 20.2 (3.0) | 18.3 (3.2) | 0.247 |
|  | Range | (15, 23) | (13, 22) |  |
|  | SE, D  Mean(SD) | 1.91 (2.13) | 2.21 (0.96) | 0.622 |
|  | Range | (-2.5, 4.75) | (0.63, 3.25) |  |
|  | BCVA, D | NA | NA | NA |
| **Surgical age:**  **2 y** |  | With follow-up | Lost to follow-up | P value |
|  | Patients | 20 | 28 | NA |
|  | Sex (M: F) | 13:7 | 14:14 | 0.302 |
|  | Age at surgery, mo  Mean (SD)  Range | 30.3 (3.9)  (24,35) | 29.0 (3.9)  (24,35) | 0.279 |
|  | SE, D  Mean (SD)  Range | 1.81 (1.76)  (-1.25,7.00) | 2.26 (2.08)  (-0.88, 7.5) | 0.248 |
|  | BCVA, D | NA | NA | NA |
| **Surgical age:**  **3 y** |  | With follow-up | Lost to follow-up | P value |
|  | Patients | 21 | 53 | NA |
|  | Sex (M: F) | 13:8 | 34:19 | 0.856 |
|  | Age at surgery, mo  Mean (SD)  Range | 40.0 (3.3)  (36,47) | 41.4 (3.5)  (36,47) | 0.121 |
|  | SE, D  Mean (SD)  Range | 1.55 (2.38)  (-5.13,8.50) | 1.25 (1.79)  (-3.00, 8.25) | 0.392 |
|  | BCVA, D | NA | NA | NA |
| **Surgical age:**  **4 y** |  | With follow-up | Lost to follow-up | P value |
|  | Patients | 22 | 34 | NA |
|  | Sex (M: F) | 14:8 | 20:14 | 0.719 |
|  | Age at surgery, mo  Mean (SD)  Range | 54.5 (3.5)  (48, 59) | 53.1 (3.1)  (49,59) | 0.887 |
|  | SE, D  Mean (SD)  Range | 0.98 (1.93)  (-3.50, 7.63) | 1.07 (1.52)  (-1.75, 5.38) | 0.793 |
|  | BCVA, D  Mean (SD)  Range | 0.47 (0.38)  (0,1.85) | 0.54 (0.50)  (0,1.85) | 0.442 |
| **Surgical age:**  **5 y** |  | With follow-up | Lost to follow-up | P value |
|  | Patients | 14 | 24 | NA |
|  | Sex (M: F) | 7:7 | 14:10 | 0.618 |
|  | Age at surgery, mo  Mean (SD)  Range | 65.0 (3.5)  (60, 71) | 66.5 (3.4)  (60, 71) | 0.191 |
|  | SE, D  Mean (SD)  Range | 0.62 (1.58)  (-2.63, 4.0) | 1.10 (1.73)  (-2.0, 4.13) | 0.205 |
|  | BCVA, D  Mean (SD)  Range | 0.37 (0.30)  (0.05, 1.30) | 0.48 (0.07)  (0.05, 1.40) | 0.176 |

Notes: #: Patients who did not complete the fourth year follow-up were defined as lost to follow-up. SE: spherical equivalent; BCVA: best corrected visual acuity; D: diopters; SD: standard deviation; M: male; F: female; y: years; NA: Not available.
